# Planning and orthodontic preparation for maxillary incisors reshaping

**DOI:** 10.1590/2177-6709.26.4.e21spe4

**Published:** 2021-09-10

**Authors:** Marcelo CASTELLUCCI, Paula MATHIAS

**Affiliations:** 1 Universidade Federal da Bahia, Departamento de Odontologia Social e Pediátrica (Salvador/BA, Brazil).; 2 Universidade Federal da Bahia, Departamento de Clínica Odontológica (Salvador/BA, Brazil).

**Keywords:** Orthodontics, Dental esthetics, Smile

## Abstract

**Introduction:**

Having a beautiful smile is the main desire of people seeking dental treatment. To achieve this goal, many variables must be considered. These include tooth alignment, color, shape and size, besides their harmonious relationship with the lips and face. An individualized diagnosis is essential to achieve the best result. Within this context, facial analysis and the characteristics of shape, size and position of maxillary incisors play a key role.

**Methods:**

This paper describes clinical situations in which interdisciplinary treatment was performed to achieve esthetic results in a conservative manner and ensuring greater stability. In most cases requiring reshaping of maxillary incisors for esthetic reasons, prior orthodontic movement is essential. The main focus of this paper is to discuss the many variables involved in these situations.

**Results and Conclusion:**

The results of treatments described in this paper were obtained by means of a multidisciplinary approach, involving Orthodontics and Esthetic Dentistry, acting in harmony and recognizing their possibilities and limitations, in order to offer patients the best esthetic solution for their smile. The best treatment option is not always the easiest or fastest. The dentist, as a health professional, should consider the patients’ wishes but also perform treatments with minimal intervention, and the best and most predictable esthetic result, also focusing on function and health.

## RESUMO

**Introdução:**

Ter um sorriso bonito é o principal desejo de quem busca um tratamento odontológico. Para se alcançar esse objetivo, muitas variáveis devem ser consideradas. Entre elas, estão alinhamento, cor, forma e tamanho dos dentes, além da sua relação harmoniosa com os lábios e a face. O diagnóstico individualizado é essencial para se alcançar o melhor resultado. Nesse contexto, a análise facial e as características de forma, tamanho e posição dos incisivos superiores têm papel primordial.

**Métodos:**

O presente artigo descreve situações clínicas em que o tratamento interdisciplinar foi realizado para alcançar resultados estéticos de modo conservador e garantindo maior estabilidade. Na maioria dos casos em que os incisivos superiores precisam ser reanatomizados por razões estéticas, a movimentação ortodôntica prévia é fundamental. O principal foco desse trabalho é a discussão das muitas variáveis envolvidas nessas situações.

**Resultados e Conclusão:**

Os resultados dos tratamentos descritos nesse artigo foram obtidos a partir de um planejamento realizado em equipe - Ortodontia e Dentística, trabalhando em harmonia, reconhecendo as suas possibilidades e limitações, são capazes de oferecer aos pacientes a melhor solução estética para o seu sorriso. A melhor alternativa de tratamento nem sempre é a mais fácil ou a mais rápida. O cirurgião-dentista, como profissional de saúde, deve acolher os desejos dos pacientes, mas realizar tratamentos com intervenção mínima e com o melhor e mais previsível resultado estético, com foco dirigido, também, à função e saúde.

## INTRODUCTION

The esthetic composition of smile involves several harmoniously arranged factors such as tooth positioning and color, harmony of shape and size of teeth to each other and with the patient’s face, relationship with the lips, facial soft tissues, bones and facial muscles.^1^ ^,^ ^2^ ^,^ ^3^

Facial analysis has become fundamental when planning orthodontic movements, orthognathic surgeries and dental rehabilitation.^3^ ^,^ ^4^ Distances and angles are often used to achieve harmonious relationships between teeth and face, especially when associating facial measurements with the dimensions of maxillary incisors, which are protagonists in smile.

The importance of maxillary incisors for the smile increases the need for interdisciplinary approaches to enable the achievement of well-positioned teeth, with shape and contour compatible with the face. Thus, the orthodontic treatment should always be in line with the Restorative Dentistry treatment, aiming at a minimally invasive approach, in search for optimum outcomes. 

Thus, the purpose of this paper is to present case reports with interdisciplinary approach, by means of Orthodontics and Esthetic Dentistry treatments, presenting situations in which the maxillary incisors required reshaping due to abnormalities of shape or size, disproportion between tooth size and face, discrepancy between bone bases and mesiodistal dimensions of teeth, emphasizing the criteria considered when planning the reshaping, as well as the most predictable treatment sequence. The paper also includes the description of care in the follow-up of treatment.

## SITUATIONS IN WHICH THE MAXILLARY INCISORS REQUIRE RESHAPING

### INCISORS WITH ABNORMALITIES OF SHAPE AND/OR SIZE

Abnormalities of shape and size are relatively common conditions in the dental clinic. Besides the third molars, the teeth most often affected by this condition are the maxillary lateral incisors^5^. Since these teeth are positioned in an important region for the smile and facial appearance, its anatomical adequacy is essential for a good esthetic outcome of any dental treatments.

**Figure 1:**
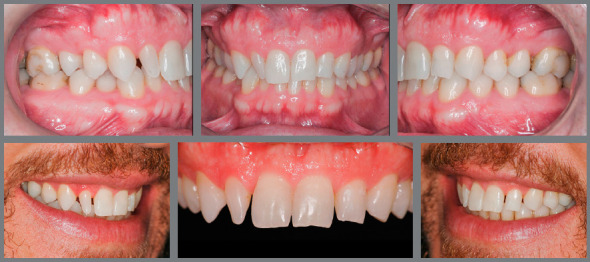
Orthodontic preparation, with space opening for reshaping of lateral incisors with abnormal shape and size.

When the anomaly in shape or size is related to smaller teeth, such as microdontia or peg-shaped incisors, reshaping with addition of material is indicated. However, since the proximal contacts are critical to the stability of mesiodistal position of teeth, it is very common that teeth adjacent to the smaller tooth have migrated, and thus the resulting space is not ideal for reshaping. Therefore, in most cases, orthodontic movement is necessary before restorations, in order to reestablish the ideal spaces, allowing harmonious reshaping of teeth (Fig 1).

### DISPROPORTION BETWEEN SIZE OF INCISORS AND FACE/SMILE

The maxillary central incisors are considered the teeth with greatest esthetic importance for the smile; thus, when planning the restorative treatment, their dimensions are frequently studied and related to the individual’s face, to allow the individualization of smile. Therefore, facial references are used to determine the ideal dimensions for each face, in both horizontal and vertical dimensions.

One facial reference often used for this purpose is the interpupillary distance, a stable facial measurement that is consolidated after the age of 14 years until adulthood.^6^ The value of this distance, divided by 6.6, represents the ideal mesiodistal dimension of the maxillary central incisor, i.e., its width. Thus, during dental rehabilitation, especially in the presence of interdental diastemas or excessively large or narrow incisors, this dimension aids the rehabilitation planning. Liao et al.^7^ mentioned the interalar distance as another reliable parameter. After analysis of 41 papers, they concluded that the apparent width of the two central incisors in combination should be equal to 70% of the interalar distance of the individual.

Another important analysis to define the size of maxillary incisors is their exposure while smiling. This exposure should range from 3⁄4 of the clinical crown of maxillary central incisors (most common in men) to exposure of 2 mm of gingiva (mostly observed in women)^8^. If this exposure is not altered by bone discrepancies or altered dimensions of the upper lip, incisal increases or orthodontic extrusion of these teeth should be considered, aiming to achieve these parameters.

### BOLTON DISCREPANCY, WITH EXCESS OF MANDIBULAR TEETH VOLUME

Bolton,^9^ when evaluating 55 pairs of dental casts of patients with occlusion close to optimal, concluded that the important factors for good occlusion include the proportion between the mesiodistal dimensions of mandibular and maxillary teeth. Thus, the author established ideal values ​​for the proportion between the sum of widths of maxillary and mandibular teeth, both for all teeth in the arch (first molar to the first molar of each arch) and for the anterior segment (canine to canine for each arch). According to their findings, the sum of widths of mandibular teeth (in the total evaluation of arches) should represent 91.3% (87.5 to 94.8%) of the sum of widths of maxillary teeth, while this proportion should be 77.2% (74.5 to 80.4%) when evaluating only anterior teeth.

**Figure 2:**
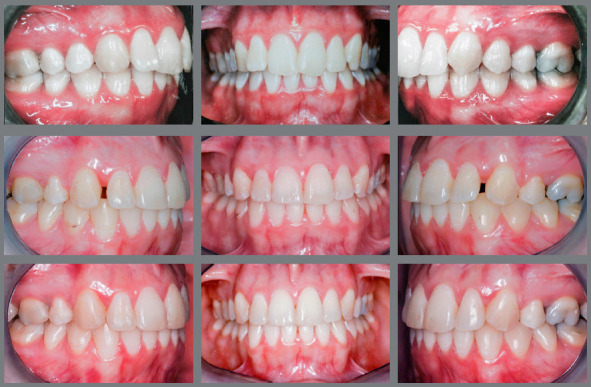
Case of Bolton discrepancy ( with excess lower tooth volume ) treated by interproximal stripping of mandibular incisors and canines, and addition of resin in the maxillary incisors and canines. Even though the maxillary teeth had adequate shape and dimension, it was necessary to reshape them to avoid excessive stripping on the mandibular teeth to compensate for the tooth size discrepancy between maxillary and mandibular teeth.

If these ratio values are greatly altered, which can be considered a “tooth size discrepancy” or “Bolton discrepancy”, adjustments are necessary to achieve good occlusion. When the result of analysis of this proportion is significantly higher than the value recommended by Bolton,^9^ it means that there is excess tooth volume in the mandibular arch in relation to the maxillary arch. If this discrepancy is not compensated, at completion of orthodontic treatment the situation will be as follows: canines in key occlusion, well related to each other, adequate overjet and remaining spaces between the maxillary anterior teeth (Fig 2). Thus, if the spaces are closed by retraction of incisors, an anterior crossbite will be created, while if they are closed by mesialization of posterior teeth, the canines will change from key occlusion to a Class II relationship. Neither of the two options should be considered. 

To solve this situation, it is necessary to adjust the proportion between maxillary and mandibular teeth by dental reshaping. Three treatment options are available for that purpose: a) the width of mandibular anterior teeth can be reduced by interproximal stripping. Then, the spaces created by stripping are closed by retraction of mandibular incisors and consequent creation of overjet, which allows closure of upper spaces by retraction of incisors; b) open (or maintain) spaces for subsequent closure by increasing the width of maxillary anterior teeth; or c) a combination of the two options.

To choose the best option, the diagnosis and planning should be individualized. During this process, several aspects must be considered, such as the relationship between enamel thickness and the amount of wear required, harmony between size/shape of incisors and face/smile, anteroposterior positioning of the lips (facial profile) and buccolingual inclination of incisors (Fig 2). These and other important aspects for planning will be discussed in more detail in this paper.

## CRITERIA TO BE CONSIDERED WHEN PLANNING THE INCISOR POSITION, BEFORE RESHAPING

### PROFILE (UPPER LIP POSITION)

One of the most important aspects for facial harmony is the volume and anteroposterior positioning of voluminous and well-positioned lips, which, besides harmony, provides beauty, attractiveness, sensuality and youthfulness to the individual^10^. Thus, this aspect should be among the most important when planning any orthodontic treatment.

The positioning of lips is markedly influenced by the anteroposterior position of incisors. The more protruding the incisors, the more protruding the lips will be. The opposite is also true. The retraction of anterior teeth tends to cause lip retraction, changing the individual’s facial profile.^10^

**Figure 3:**
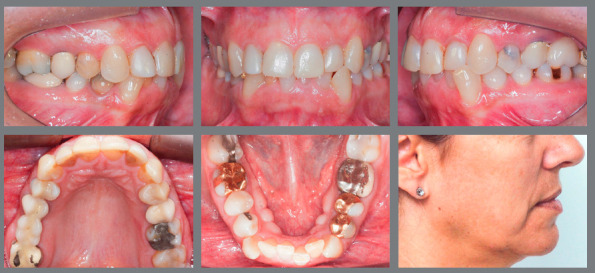
Bolton discrepancy, with excess lower tooth volume and retracted upper lip. Indication of protrusion and addition of restorative material in the maxillary incisors, to harmonize the proportion between maxillary and mandibular incisors and increase the upper lip volume.

**Figure 4:**
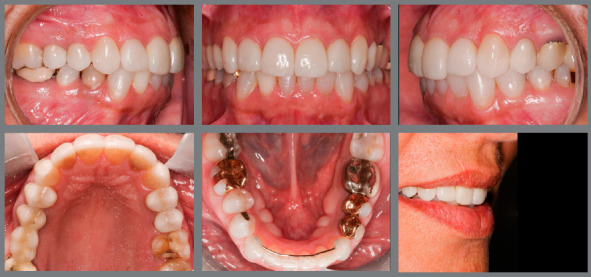
Incisor projection (associated with interproximal stripping of mandibular anterior teeth) to dissolve the mandibular crowding, and subsequent reshaping of maxillary incisors with ceramic veneers, and lip volume harmonization.

When assessing the planning of these cases, a question may arise: what is the relationship between the anteroposterior position of incisors and their mesiodistal reshaping? There is a direct relationship. When there is need to open spaces to increase the width of incisors, the teeth are protruded to occupy a more external arch and consequently more space, increasing the lip projection. Conversely, when the treatment option for Bolton discrepancy is interproximal stripping for future incisor retraction, one of the likely consequences is lip retraction. Thus, generally speaking, in a case of disproportion between the size of maxillary and mandibular teeth, if the patient has retruded lips, the treatment with space opening for posterior augmentation of teeth with restorative material is the most indicated (Figs 3 and 4). Conversely, if the lips are excessively protruded, the treatment with interproximal stripping and subsequent retraction of incisors should be chosen.

### INCLINATION OF INCISORS

If the anteroposterior position of maxillary incisors is directly related to the positioning of lips and consequently to the patient profile, the buccolingual inclination of these teeth has an important influence on the appearance of incisors during speech and smile. The characteristics of human vision lead the perception of the size of an object to occur according to the dimensions of the part of that object that reflects light horizontally.^11^ As a result, when the incisors present excessive buccal tipping, their apparent height is smaller than the actual one, since part of the light on the buccal faces of these teeth is deflected upwards.

Thus, this aspect should also be evaluated when planning the reshaping of anterior teeth. When opening of maxillary anterior spaces is necessary to increase the width of incisors, there is a tendency of increased buccal tipping of their crowns. When the incisors are too upright or lingually tipped, this is favorable. However, when the initial inclination of these teeth is adequate and due to limitation of buccal bone thickness, bodily tooth protrusion cannot be performed (crown and root proportionally buccally tipped), this movement can lead to a situation in which the incisors would have an apparent length smaller than their actual length. Besides, the glare resulting from light reflection on the buccal surfaces would be displaced from the center to the incisal third,^12^ assigning these teeth an unpleasant appearance,^13^ ^,^ ^14^ making the smile less attractive.

**Figure 5:**
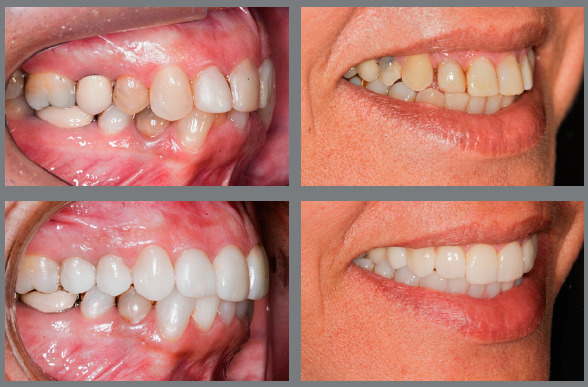
Ceramic veneer, compensating the excessive buccal tipping resulting from the projection of maxillary incisors.

In these situations, the advantages and disadvantages of each treatment option should be evaluated. When, after careful evaluation, it is concluded that the spaces must be opened for tooth augmentation, even if their resulting buccolingual inclination is not ideal, it is possible to minimize this disadvantage during restorative treatment. For that purpose, the thickness of restorative material can be increased in the cervical third of incisors in relation to the incisal. In the case of restorative treatment planning, some situations can be considered: 1) When there is no need to increase the tooth length, the thickness of restorative material on the tooth can be increased, especially in the cervical third of incisors, in relation to the incisal; and 2) When there is a need for incisal augmentation of these teeth, they can be restored with material on the entire buccal surface (veneer-type restoration), performing a slight incisal augmentation, in which this incisal third assumes a steeper inclination toward the palate, eliminating the appearance of buccal projection from the incisal edges of maxillary anterior teeth. In situations where this strategy is indicated, the material of choice can be resin, or even dental ceramics, since they allow for more efficient manipulation of the inclination of buccal surfaces of incisors (Fig 5).

### HEIGHT/WIDTH RATIO AND TOOTH SHAPE

For planning additions of material in anterior teeth, either due to the need to close mesiodistal spaces or increase the length, the dental dimensions must not only be related to the patient’s face, they should always be proportional to each other, i.e., the width/height ratio of each tooth must guide the planning of addition of restorative material, avoiding excessively wide or narrow teeth or even very long or very short.

The mean width/height ratio of central incisors, the teeth most apparent in smile, is 78 to 86%, i.e., the tooth width corresponds to approximately 78 to 86% of its height.^15^ ^,^ ^16^ Studies that assess and consider factors such as more recurrent dental esthetic disposition establish that this ratio should ideally be 78%,^17^ leaving the central incisor slightly narrower and thus more elongated.

After defining the dimensions of central incisors, the lateral incisors and canines should be addressed. The width/height ratio of lateral incisors should be from 76 to 79%, and 77 to 81% for the canines. Also, in frontal view, the apparent width of the six anterior teeth should decrease from the midline to the posterior region, so that the canine appears to be 70% of the width of the lateral incisor and this, in turn, should appear to have a width corresponding to 70% of the central incisor.^16^ ^,^ ^17^

If it is necessary to close diastemas with restorative material (Bolton’s discrepancy), the orthodontist must maintain an integrated plan with the restorative dentist, so that they can measure the teeth, especially their width, to calculate the amount of material that will be added to each individual tooth and the location where this material will be added, so that the dimensional balance of teeth is not altered.

Regardless of the option chosen by the patient and the dentist, it should be considered that the apparent width and height of teeth can be altered by many factors, from positioning of soft tissue, such as the lips, to the primary and secondary anatomy of teeth, addressing the areas of light reflection, shadow and areas of transition between teeth.

**Figure 6:**
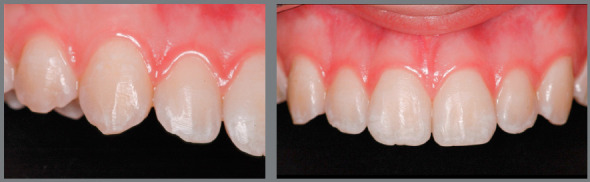
Detail of the shape of proximal surfaces of anterior teeth: straight mesial surfaces of central incisors, and convex distal surfaces of lateral incisors and mesial surfaces of canines.

Besides the dimensional aspects of teeth, their shape and proximal aspects should be considered when planning dental reshaping. The closure of diastema in the interdental midline region, between the two maxillary central incisors, involves the need for additions on their mesial surfaces, which are usually flatter, i.e., with less convexity, complicating the shaping of composite resin without the presence of cervical excess. Conversely, the convexity of distal aspects of lateral incisors and mesial surfaces of canines facilitates the insertion of restorative material to close the interdental space in a more anatomical manner, with the use of a smaller amount of material in the cervical area, facilitating the technique and avoiding cervical excesses of restorative material (Fig 6).

### VERTICAL POSITIONING OF INCISORS

Most aspects considered in this paper concern issues involving the planning of changes to the mesiodistal dimensions of incisors. However, as described in the previous item, the length of these teeth is as important for the esthetic result of treatment as their width - in some situations, even more.

**Figure 7:**
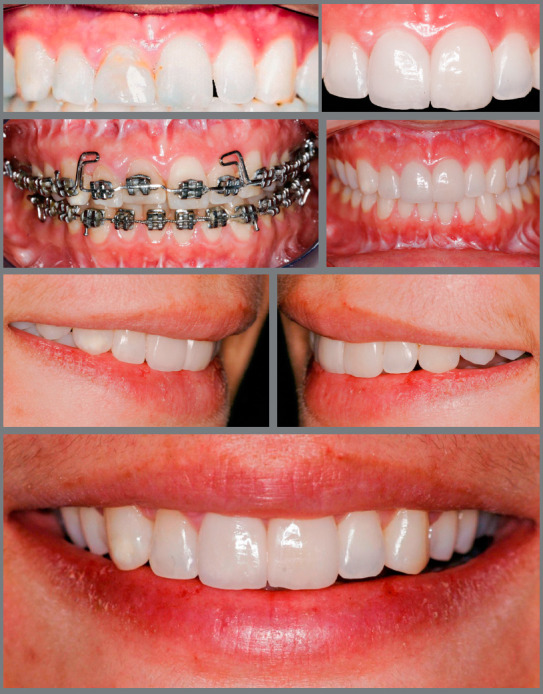
Intrusion of tooth #11, to level the gingival margins and later reshape the incisors with ceramic veneers.

However, when planning dental positioning prior to the increase in height, fewer variables need to be evaluated. Two of them are the most important:

» Overjet - When the maxillary incisors require elongation by the addition of restorative material, some overjet should be left, so that there is adequate space for sufficient thickness of restorative material to have the required strength. Thus, in these cases, depending on the patient’s occlusion, a small retraction of mandibular incisors may be necessary, or a small projection of maxillary incisors.
» Periodontal aspects - In some situations, the height of incisors is asymmetrical, either because of tooth wear or fractures or because of asymmetry in the gingival contour (Fig 7). In these cases, it is very important to identify the reason for the lack of gingival leveling. If the cause is excess gingival tissue in one or more incisors, the most appropriate approach is periodontal surgery, so that the excess tissue is removed, and the gingival contour becomes symmetrical and harmonious. However, if the asymmetry occurs due to passive extrusion of a tooth whose incisal third is worn or fractured, the best alternative is harmonization of the gingival contour by orthodontic intrusion of the extruded tooth.^18^ This movement will result in unleveling of the incisal edges between the intruded tooth and adjacent teeth, which will require correction by an esthetic restorative procedure (Fig 7).

In these cases, the advantage of intrusion compared to periodontal surgery is that the incisors will have balanced mesiodistal dimensions in the middle and cervical thirds, which will not occur if they are kept in position, due to the divergent shape of their crowns in incisal direction.

Therefore, a detailed periodontal examination is very important, with measurement of probing depth and evaluation of imaging exams, to reach a correct diagnosis and proper planning.

### RESTORATIVE MATERIAL TO BE USED

For the addition of restorative material aiming to change the mesiodistal dimension of teeth, the positioning of interdental spaces that will be rehabilitated is directly related to the size of teeth (as aforementioned) and also to the restorative material that will be used. In the case of esthetic additions, two restorative materials can be considered: dental ceramics and composite resins.

Dental ceramics is a material widely used in restorations, such as veneers or dental crowns. The indication of this material is usually associated with the need for dental preparations that enable the creation of an insertion axis for the ceramic restoration, based on the presence of expulsive areas in the teeth. Since ceramic restorations usually involve the entire buccal surface of teeth, planning their location and shape is strongly associated with tooth dimensions and planning of the esthetic design of smile.

Composite resin is a very versatile material and can be used for partial restorations or for larger restorations - such as the entire buccal surface of teeth, for example. Besides, it is the material of choice for small additions, such as in cases of diastema closure, small shape changes and partial reconstructions, and for use in younger patients.

Despite being an esthetic restorative material, it has color instability, especially in the marginal region, when exposed to the oral environment^19^ and to some substances containing dyes.^20^ This change in the color of material is one of the main reasons for the replacement of restorations, especially in anterior teeth.^21^ Therefore, when small additions of composite resin are used to close spaces, they should be used in less critical esthetic areas, such as the distal surface of lateral incisors, avoiding more medial regions of the smile, which are more relevant from an esthetic point of view.

**Figure 8:**
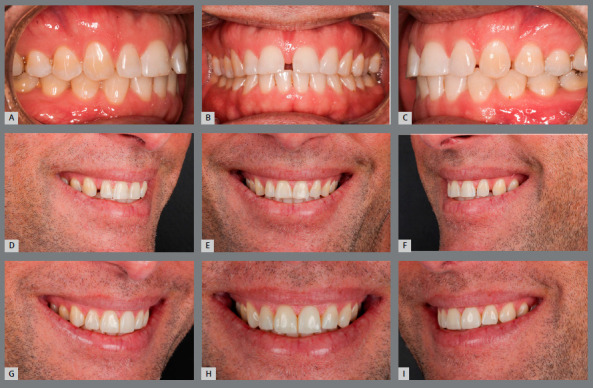
Closure of midline diastema (**A**-C) by means of an interdisciplinary approach. The maxillary incisors were mesialized, to orthodontically close the diastema, displacing the spaces to the posterior region (**D**-F), avoiding the need to insert restorative material in the mesial surfaces of these teeth. To coincide the upper dental midline with the facial midline, teeth #11 and #12 were moved more than teeth #21 and #22. Then, the spaces were closed by adding composite resin on the distal surfaces of lateral incisors and mesial surfaces of canines (**G**-I). The central incisors were increased vertically to provide a better height/width ratio, a more harmonious smile arc and adequate overbite.

In the clinical case illustrated in Figure 8, the presence of a broad central diastema is observed. When planning the closure of this diastema, some factors were considered, such as: 1) dimension of maxillary incisors (the central incisors were very large); 2) location of the diastema (central diastema); and 3) restorative material used (composite resin, chosen in agreement with the patient, due to the desire to avoid tooth stripping). Thus, due to the combination of these factors to close the diastema, it was necessary to achieve a better distribution of interproximal spaces, aiming at the esthetic balance of smile and the durability of composite resin restorations. For that purpose, the central space was shifted to the right and left sides, and positioned between the lateral incisors and canines. After tooth movement and stabilization of movement, the teeth were bleached, and spaces were closed. The central incisors were restored to re-establish the height/width ratio, esthetic composition of smile and adequate overbite.

## MORE ADEQUATE SEQUENCE OF PROCEDURES

### DIAGNOSIS

Any interdisciplinary treatment requires an integrated planning. In the specific case of orthodontic treatment of a patient who will undergo some type of esthetic reshaping of maxillary incisors, besides the diagnosis and planning for correction of occlusal and facial problems, as in any other case, it is necessary to carefully evaluate all aspects discussed in the previous item, to determine the final position of teeth that will be reshaped, as well as the adjacent and antagonist teeth.

What will be the final dimensions of incisors? Which restorative material will be used? In which regions will spaces be left? What will be the dimensions of these spaces? And in vertical direction? Should any incisors be intruded or extruded? These are the questions to be answered before treatment is initiated. For that purpose, all professionals involved - orthodontist, restorative dentist and periodontist - must participate in the diagnosis and planning process.

### PLANNING

**Figure 9:**
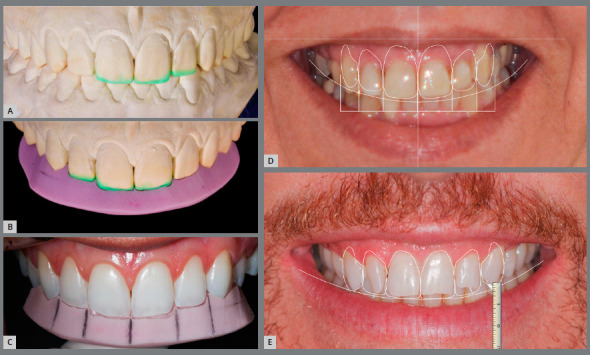
Waxing (**A, B,** C) and digital smile planning (**D,** E) before incisor reshaping.

To achieve greater predictability, there are tools for simulation of results that are fundamental to test the initially planned results. This phase allows an anticipated view of the results that will be achieved, which allows adjustments to the planning even before treatment onset. This procedure can be performed at various times during treatment, yet it should be done during initial planning and at the time between orthodontic finalization and accomplishment of restorative procedures. For this, there are two options: diagnostic wax-up and digital smile planning (Fig 9).

### TOOTH MOVEMENT

**Figure 10:**
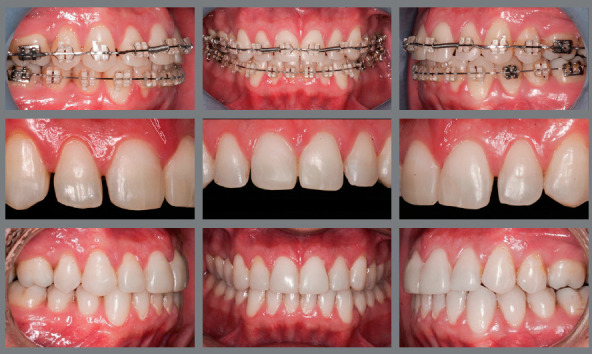
Confirmation of the final position of teeth, with the appliance still in place, to allow for any adjustments still necessary at that moment.

After planning the final position of teeth that will be reshaped, as well as of adjacent and antagonist teeth, the orthodontist should start orthodontic treatment to achieve the objective outlined in the planning phase. This paper does not aim to discuss the types of appliances or orthodontic mechanics; however, regardless of the strategy used by the orthodontist, after reaching the initial objective, further exams should be conducted to confirm if that configuration is the most suitable to achieve the best esthetic results as possible. At this moment, a new planning, either digital or conventional (diagnostic wax-up) should be performed. There are some cases in which the initial position of teeth does not allow for a very accurate simulation of the final configuration of smile, which becomes possible with the new tooth positions. It is very important to perform this evaluation with the orthodontic appliance in place (if brackets are used), so that small adjustments shown to be necessary can still be made (Fig 10). 

### BLEACHING

Almost all patients submitted to reshaping of incisors for esthetic reasons want these teeth to be lighter, in addition to larger. Thus, most of them undergo bleaching. Traditionally, the orthodontic appliance (brackets or attachments) is removed before bleaching, which leads to the need for special care with retention during this period. However, it is possible to perform bleaching with the appliance still in place.

Since the bleaching procedure takes 21 to 28 days in the average and is usually performed soon after completion of orthodontic treatment, this period is critical in relation to retention. Therefore, bleaching with orthodontic devices still in place becomes interesting, thus allowing the stability of results of orthodontic movement. Studies show that the presence of brackets does not compromise the results of bleaching, maintaining the homogeneity of bleaching along the entire buccal surface of teeth.^22^ ^,^ ^23^

**Figure 11:**
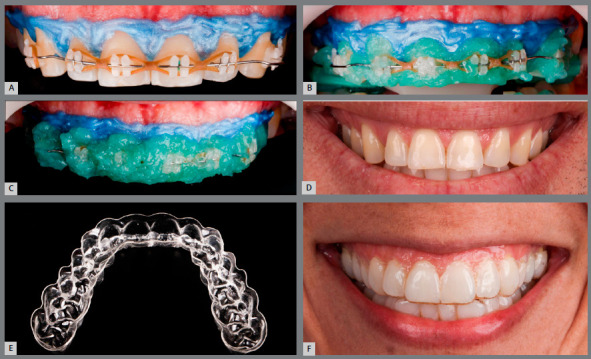
Bleaching performed during orthodontic treatment with brackets (**A**-D) and aligners (**E**, F).

Despite this advantage, bleaching techniques for patients still using orthodontic brackets are limited to bleaching techniques performed in the office or at-home techniques using pre-loaded trays. The conventional at-home technique cannot be used, due to the impossibility of making customized trays in these cases. It should be mentioned that orthodontic patients using aligners (Figs 11E and 11F) can perform at-home bleaching using their own aligners.^24^

Figures 11A to 11C show a patient shortly after orthodontic movement and still with the brackets in place, aiming to stabilize the movement, in which in-office bleaching was chosen with 37% hydrogen peroxide (Whitness Automix, FGM ) used in three clinical sessions, with seven-day intervals between them. In Figure 11D, it is possible to see the harmonious and homogeneous color result obtained after in-office bleaching in a patient during orthodontic treatment.

### REMOVAL OF ORTHODONTIC APPLIANCE

After confirming the adequacy of position of all teeth, from an esthetic and functional point of view, the orthodontist should remove the orthodontic appliance and refer the patient to the restorative dentist. This is another moment that requires the integration between professionals involved. Since planning is done very precisely, any modification in the position of teeth can impair the result. Therefore, the time between removal of orthodontic appliance and restorative treatment should be as short as possible. To minimize risks, ideally, both procedures should be performed on the same day. For this, the professionals involved in treatment must work together and in harmony, not only with regard to the technical aspects of planning, but also with regard to their scheduling.

### RESTORATIONS

Dental restorations performed on anterior teeth usually involve changes in shape, volume or dimensions of teeth, aiming at the harmonious composition of smile and the relationship between smile and face. For this, the entire project to change these teeth must be previously performed from photographs, digital plans, followed by the creation of prototypes, either by wax-ups or diagnostic models (Fig 9). This plan is shared with the patient by a test in mouth, known as a mock-up. In this test, a temporary product (bis-acrylic resin) is inserted to the patient’s teeth, simulating the final treatment result, following a mold previously made from molding of the prototype. After approval by the patient of the design project for their teeth or for the composition of their smile, the actual restorations are performed.

Esthetic restorations are adhesive and should prioritize planning with minimal intervention, without unnecessary wear of teeth and respecting the masticatory function and occlusal balance. The restorative materials used for this purpose are composite resins and dental ceramics, and must be used following careful and detailed techniques - which include isolation of the operative field, judicious application of adhesive and light-curing protocols, handling of restorative material, occlusal adjustments, finishing and polishing, to ensure the longevity of esthetic restorations. Maintenance and monitoring programs for these restorations should be included in the patient’s treatment.

## CARE REQUIRED DURING RETENTION AND FOLLOW-UP

Any tooth that is moved tends to return to the initial position, because of the “memory” of the periodontal ligament.^25^ Therefore, all patients undergoing orthodontic treatment must go through a retention period, in which appliances are placed aiming at maintaining the results achieved during treatment. The factors that contribute to the stability of tooth position after treatment include the use of retainers, the integrity of periodontium and the ratio of teeth with their antagonists (adequate intercuspation) and adjacent teeth (proximal contact points).

**Figure 12:**
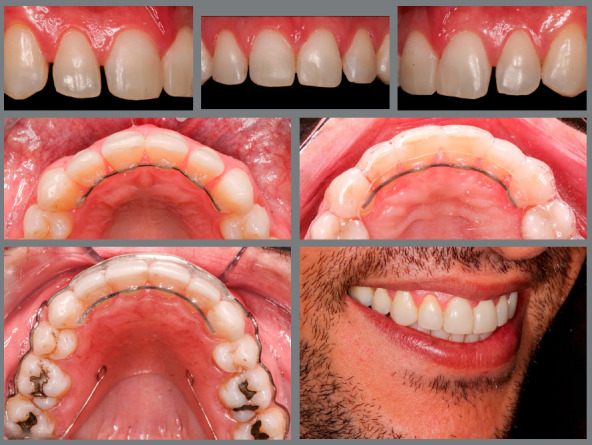
Fixed lingual retainer associated with removable appliance, for maintenance of incisor position during the period between removal of orthodontic appliance and accomplishment of restorations.

When the incisors are prepared for reshaping by the addition of restorative material, some of them are left without interproximal contacts until the restorations are placed, which can compromise the stability of their position, especially in mesiodistal direction. Therefore, these teeth should be kept in fixed retention until restorations are performed. This is essential to achieve the planned result. Any small relapse can compromise the space needed for the teeth to have perfectly proportional dimensions and, even worse, prevent the adaptation of ceramic restorations (whether crowns or laminates), which would cause a big waste of time and money, besides patient dissatisfaction. For esthetic reasons, these retainers can be placed on the lingual surfaces of these teeth and adjacent teeth (Fig 12). Besides, to minimize the risks, the time between removing the appliance and performing restorative procedures should be as short as possible.

After completion of the restorative stage of treatment, new retainers must be made, so that they adapt to the new dental and occlusal condition. In cases of extensive ceramic restorations, the use of acrylic occlusal plates should be considered, mainly in patients with history of bruxism, so that the restorations are preserved.

## FINAL CONSIDERATIONS

It seems positive that we are living a moment in which esthetics is highly valued by everyone; after all, self-esteem is fundamental for people to feel good, and well-being is directly linked to health. However, it is also a time when everything has become urgent and there is a rush to solve any issue - and this is not different for dental treatments. When there is more than one treatment option, many people prefer the faster alternative; however, concerning esthetic aspects, especially involving the appearance of maxillary incisors and the smile, the quicker option may not be the most adequate or the most conservative. Many rotated or crowded incisors have been unnecessarily stripped and their remnants covered with restorative material, while orthodontic treatment alone or in combination with esthetic restorative treatment would be a more conservative treatment option.

Conservative treatments tend to provide better, more natural, longer lasting results with fewer adverse side effects. Mutilating treatments can cause irreversible damage to the esthetics of smile and oral health of patients. Above all, the dentist is a health professional and must work as such, respecting the particularities of each case, accepting the wishes of patients and always proposing to perform the most conservative treatment with the best esthetic result as possible, never deviating from the better for the patient function and health.
